# Disinfection effect of microwave radiation on Bacillus subtilis as indicator organism on contaminated dental stone casts under dry and wet conditions

**DOI:** 10.3205/dgkh000294

**Published:** 2017-08-08

**Authors:** Mahmood Robati Anaraki, Shiva Mahboubi, Tahereh Pirzadeh, Farzaneh Lotfipour, Naimeh Torkamanzad

**Affiliations:** 1 Dental and Periodontal Research Center, Tabriz University of Medical Science, Tabriz, Iran; 2 Department of Prosthetic Dentistry, Dental School, Tabriz University of Medical Science, Tabriz, Iran; 3 Department of Prosthetic Dentistry, Dental School, Kurdistan University of Medical Science, Sanandaj, Iran; 4 Department of Microbiology, Medical School, Tabriz University of Medical Science, Tabriz, Iran; 5 Faculty of Pharmacy, Tabriz University of Medical Sciences, Tabriz, Iran; 6 Dental School, Tabriz University of Medical Science, Tabriz, Iran

**Keywords:** Bacillus subtilis, disinfection, stone cast, microwaves, moist and dry conditions

## Abstract

**Objective:** The disinfection of dental stone casts using microwave radiation has been shown, but doubts remain regarding its efficacy under various conditions. The aim of the present study was to evaluate the efficacy of microwave disinfection on wet and dry dental stone casts contaminated by a resistant microorganism.

**Material and methods:** In this in vitro study, 34 stone half-casts were prepared, contaminated with *Bacillus subtilis* and divided into two groups. After drying the specimens of one group for 15 minutes using 450 W microwave energy, all the wet and dry specimens were exposed to 900 W microwave energy for 5 minutes. Specimens were then individually transferred to nutrient broth culture medium and after 10 minutes, one milliliter from each tube was cultured in nutrient agar media for 24 hours, and the colonies were counted in CFU/mL. Data were analyzed using multifactorial ANOVA and Bonferroni tests.

**Results:** Casts in both wet and dry groups were disinfected to a high level (6 log), with no statistically significant differences between them (P<0.05).

**Conclusion:** According to the results, microwave irradiation can disinfect dental stone casts to a high degree, irrespective of moisture level. However, the result should be confirmed by exploring with other species of resistant microorganisms.

## Zusammenfassung

**Zielsetzung:** Obwohl die Desinfektion von Dentalabgüssen mit Mikrowellen bereits gezeigt werden konnte, bestehen Zweifel hinsichtlich der Wirksamkeit unter verschiedenen Bedingungen. Daher sollte die Wirksamkeit vergleichend auf nassen und trockenen Dentalabgüssen nach Kontamination mit *Bacillus subtilis* in vitro geprüft werden.

**Material und Methode:** 34 Zahnabgüsse wurden präpariert, mit *B. subtilis* kontaminiert und in zwei Gruppen aufgeteilt. In der ersten Gruppe wurden die Prüfkörper mit 450 W Mikrowellenenergie für 15 min getrocknet. Danach wurden die getrockneten und parallel die feucht belassenen Prüfkörper für 5 min mit 900 W Mikrowellenenergie exponiert, für 10 min in Nährbouillon überführt, davon 1 ml auf Agar ausplattiert und nach 24 h die Kolonien gezählt. Die Daten wurden nach mehrfaktorieller Varianzanalyse mittels ANOVA mit dem Bonferroni-Test analysiert.

**Ergebnisse:** In beiden Gruppen wurde eine Abtötung um 6-log ohne signifikanten Unterschied erreicht (P<0.05).

**Schlussfolgerung:** Mittels Mikrowellen (900 W) können Dentalabgüssen, unabhängig ob sie trocken oder feucht sind, desinfiziert werden. Allerdings sollte das Ergebnis durch Prüfung mit weiteren Testspezies untermauert werden.

## Introduction

Microwave disinfection has been shown to be a viable alternative technique to chemical disinfection, and many resistant microorganisms that cannot be eliminated even with longer contact times in disinfectant solutions can be effectively disinfected using a microwave oven [1], [2], [3]. In the case of contaminated stone casts, evidence shows that microwave irradiation at energy levels of 800–900 W for 5 minutes is also an effective disinfection method. Despite the inherent weakness and sensitivity of stone casts to loss of moisture [4], microwave radiation has little adverse effect on physical and mechanical properties of the stone [5], [6].

In order to recommend the use of microwave irradiation as an effective disinfection technique based on EN 1040 standards [7], it is necessary to evaluate the effect of 900 W microwave irradiation as a safe energy level against an optimum indicator bacteria such as *Bacillus subtilis* [8], along with the effect of wetting or drying of casts on disinfection efficacy. It was shown previously that in the case of contamination with *B. subtilis*, the stone cast could only be disinfected (but not sterilized) in a dry condition using a microwave energy level of 850 W for 10 minutes. The cast was sterilized only when immersed in water or NaCl solution while applying the same energy level and duration of irradiation [9]. This protocol, however, has detrimental effects on the dimensional accuracy and surface smoothness of the casts. Meanwhile, there is evidence showing no significant difference between dry and moist specimens in terms of eliminating non-indicator microorganisms at a 600 W energy level [10]. It seems that the testing conditions may have considerably influenced the outcomes of these studies, and deleterious boiling conditions may not necessarily be required for effective disinfection of the dental stone casts, which is enough prior to sterilization [7]. 

The present study was designed to evaluate the efficacy of microwave irradiation in adequately disinfectioning stone casts contaminated by indicator bacteria under wet and dry conditions.

The hypotheses of the present study were:

1. Microwaves irradiation with 900 W energy levels can effectively disinfect casts contaminated with *B. subtilis*.
2. Wetting or drying of casts has no effect on the quality of disinfection.

## Materials and methods

### Pilot study

A pilot study was prepared to explore the effective energy level and exposure duration of microwave irradiation. For the experiment, spherical stone specimens 10 mm in diameter (n=205) were separated from top of the alveolar ridge of a stone dental cast which had spherical beads in the place of teeth. A model was prepared by adhering 10 similar plastic spherical beads on the alveolar crest of an edentulous dental arch model made of wax, converting it to an acrylic cast by the conventional flasking technique used for resin acrylic dentures, and duplicating the model by making a conventional irreversible hydrocolloid impression and pouring with dental stone type III. All procedures in the pilot and main experiments were carried out in a sterile and microbiologically safe manner: bowls, mixing spatulas and impression trays were disinfected with 70% ethanol, and tweezers, pincers, and wax knives were flame sterilized when necessary. 

The stone specimens were inoculated by soaking in broth cultures containing 10^7^–10^8^ CFU/mL *B. subtilis* (ATCC 5127) for 10 min, except for 2 negative control specimens. The specimens, after removing from broth, were divided into 20 study groups of specimens, each for different irradiation durations and irradiation powers with 3 specimens as positive controls. The irradiation of the experimental groups was carried out at energy levels of 300, 450, 600 and 900 W for 5, 7, 10, 15 and 20 minutes after the specimens were allowed to absorb the appeared broth on surface. After treatment and cooling, each of the experimental and control specimens were placed into separate tubes of nutrient broth for 10 minutes, and 1 mL aliquot from each tube was cultured in nutrient agar for 24 hours at 37°C. Colonies were counted as CFU/mL. The results were analyzed using multifactorial ANOVA and Bonferroni tests.

### Main study

The dental stone cast specimens were fabricated by pouring silicone molds of a maxillary dental arch. Type IV stone (Elite Model, Zhermack, Italy) was used to pour 17 casts in a sterile manner based on manufacturer’s instructions. The casts were separated into halves before final setting, using a sterile stone knife for simple separation after hardening. 

**Figure 1:**
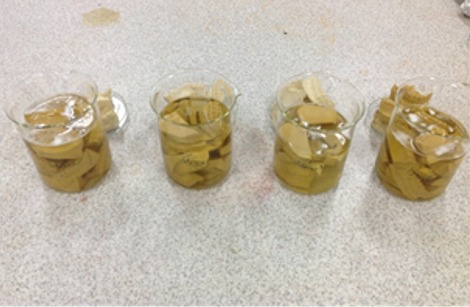
Inoculation of stone casts with suspension of *Bacillus subtilis*

A standard strain of *B. subtilis* (ATCC 6633; provided by the Pasteur Institute, Tehran, Iran) was used in the present study, and a microbial suspension (10^7^–10^8^ CFU/mL) was prepared to contaminate the stone casts. The half-cast specimens were immersed in the microbial suspension for 10 minutes, except for one negative control specimen, which was included in the study without contamination to validate the sterility of the study procedures (Figure 1 (Fig. 1)). After the specimens were retrieved from the suspension, they were randomly divided into two study groups of n=15 each. 

**Figure 2:**
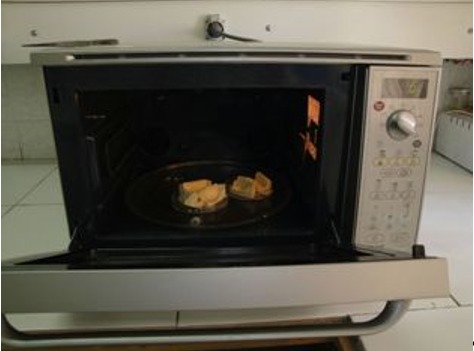
Microwave set with inoculated stone casts prepared for irradiation

The specimens in one group were dried in a microwave oven (Samsung PG 3210, China) for 15 minutes at 450 W and a frequency of 2450 MHz. Then they were irradiated at 900 W for five minutes, similar to the wet group. The wet specimens were irradiated immediately after removal from the broth tubes and absorption of their surface liquid (Figure 1 (Fig. 1) and Figure 2 (Fig. 2)).

**Figure 3:**
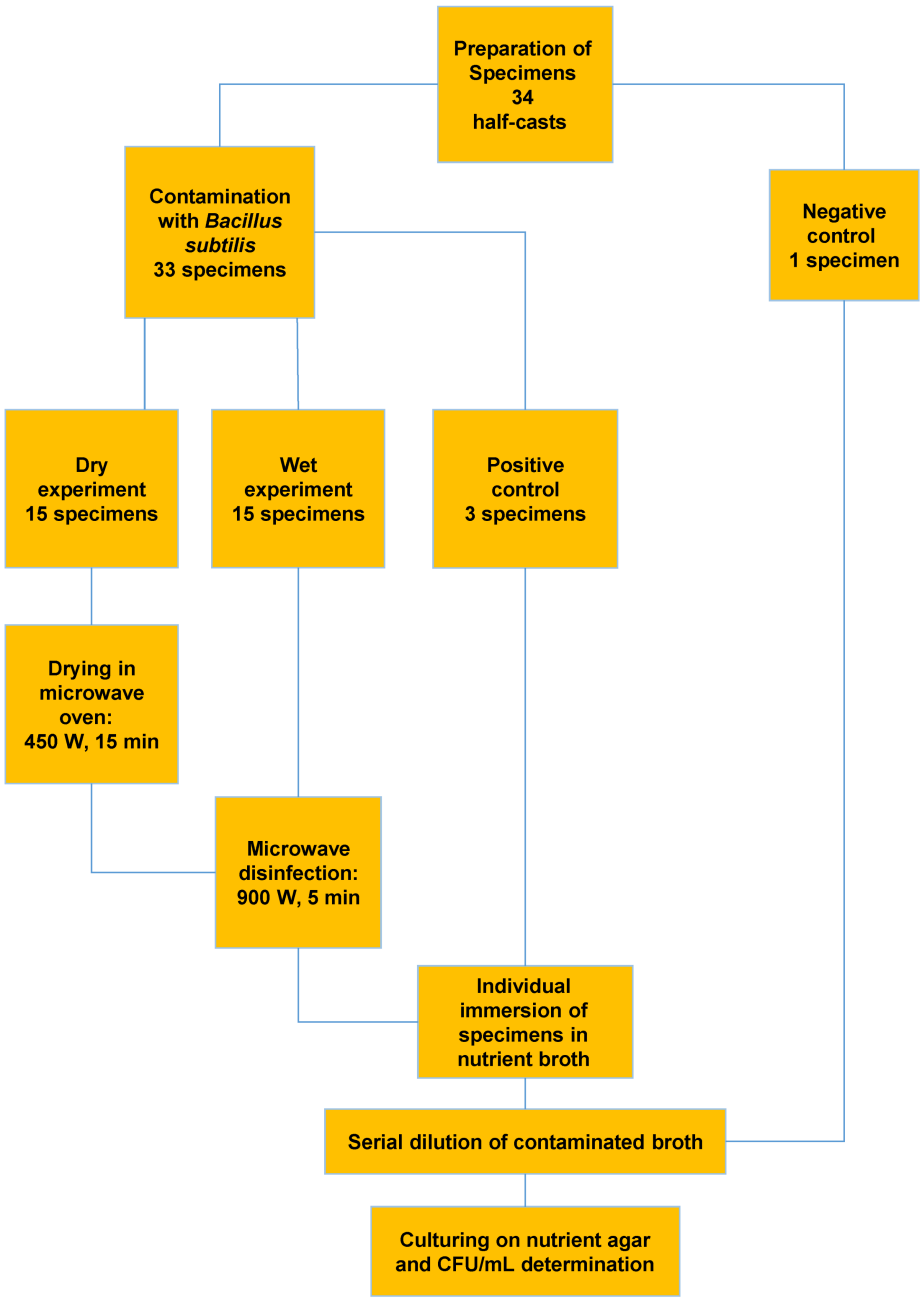
Design of main experiment

Three positive control specimens were selected with contamination and without sterilization to confirm the vitality of microorganisms, the validity of the contamination the casts, and to determine the initial counts of microorganisms on the study specimens. After irradiation, each specimen was placed in a sterile beaker containing 100 mL of sterile nutrient broth (34 beakers total). The beakers were shaken with great care, and after 10 minutes, 0.01 mL aliquots were separately cultured on agar plates. To increase the accuracy of initial colony counts, contaminated broth was serially diluted from 10^7^–10^1^ CFU/mL. All the plates were incubated at 37°C for 24 hours and *B. subtilis* colonies were counted in terms of CFU/mL (Figure 3 (Fig. 3)). The results were analyzed using ANOVA and t-tests.

## Results

### Pilot study

**Figure 4:**
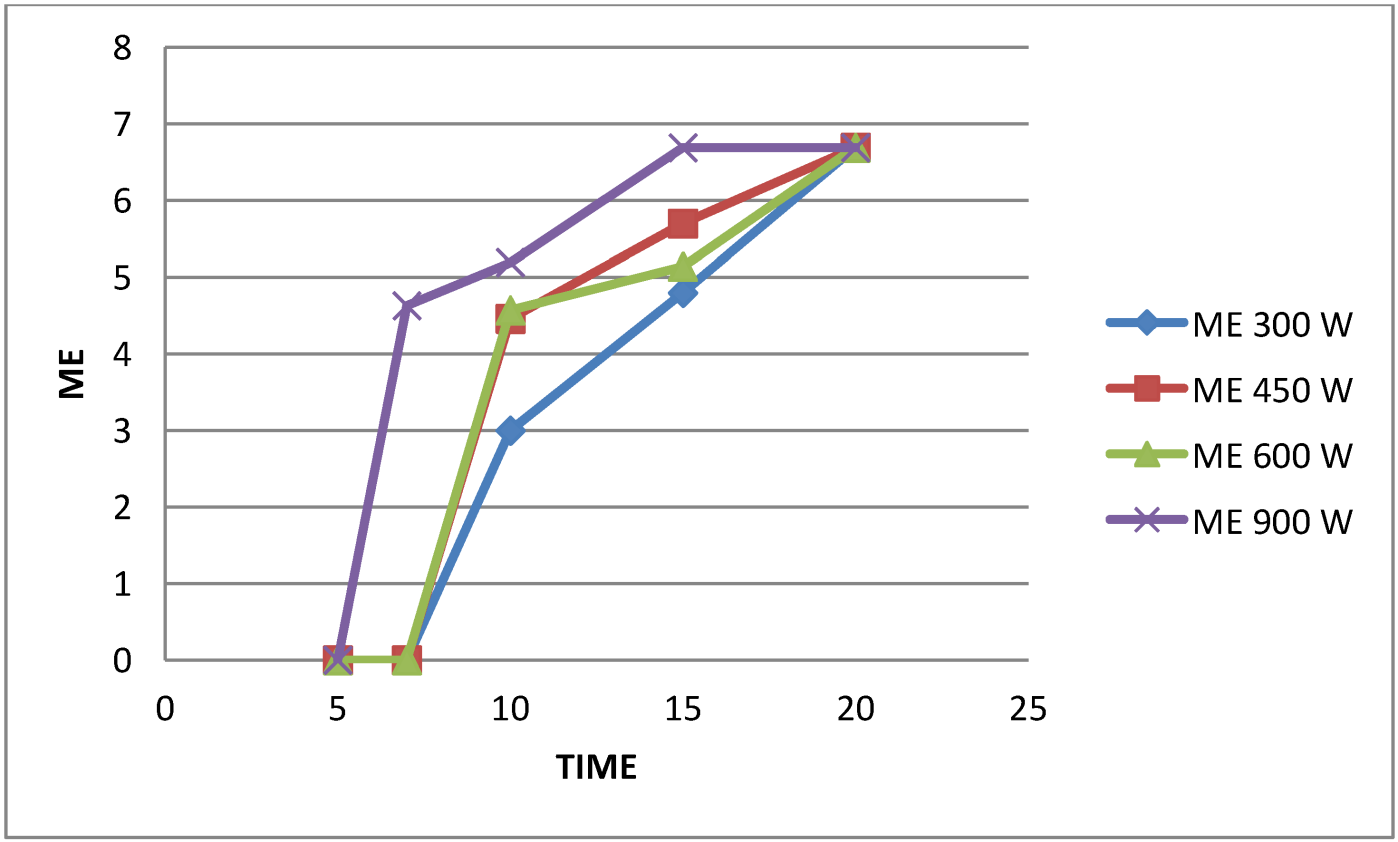
Microbicidal effect (ME) of microwave on *Bacillus subtilis* at different energy levels and irradiation times

Table 1 (Tab. 1) and Figure 4 (Fig. 4) show that applying microwaves for 20 minutes resulted in 6-log reductions in viability on contaminated specimens, regardless of the energy level, but when irradiation duration was reduced to 15 minutes, 900 W were necessary to achieve this level of disinfection. The microbicidal effect (ME) in Table 1 (Tab. 1) was calculated by subtraction of the log_10_ value of the counts after microwave treatment from the log_10_ value of the counts without exposure to the disinfection procedures (the positive control). Energy level and irradiation duration produced significant effects individually and interacting with each other (P<0.001) (Data not shown).

### Main study

**Figure 5:**
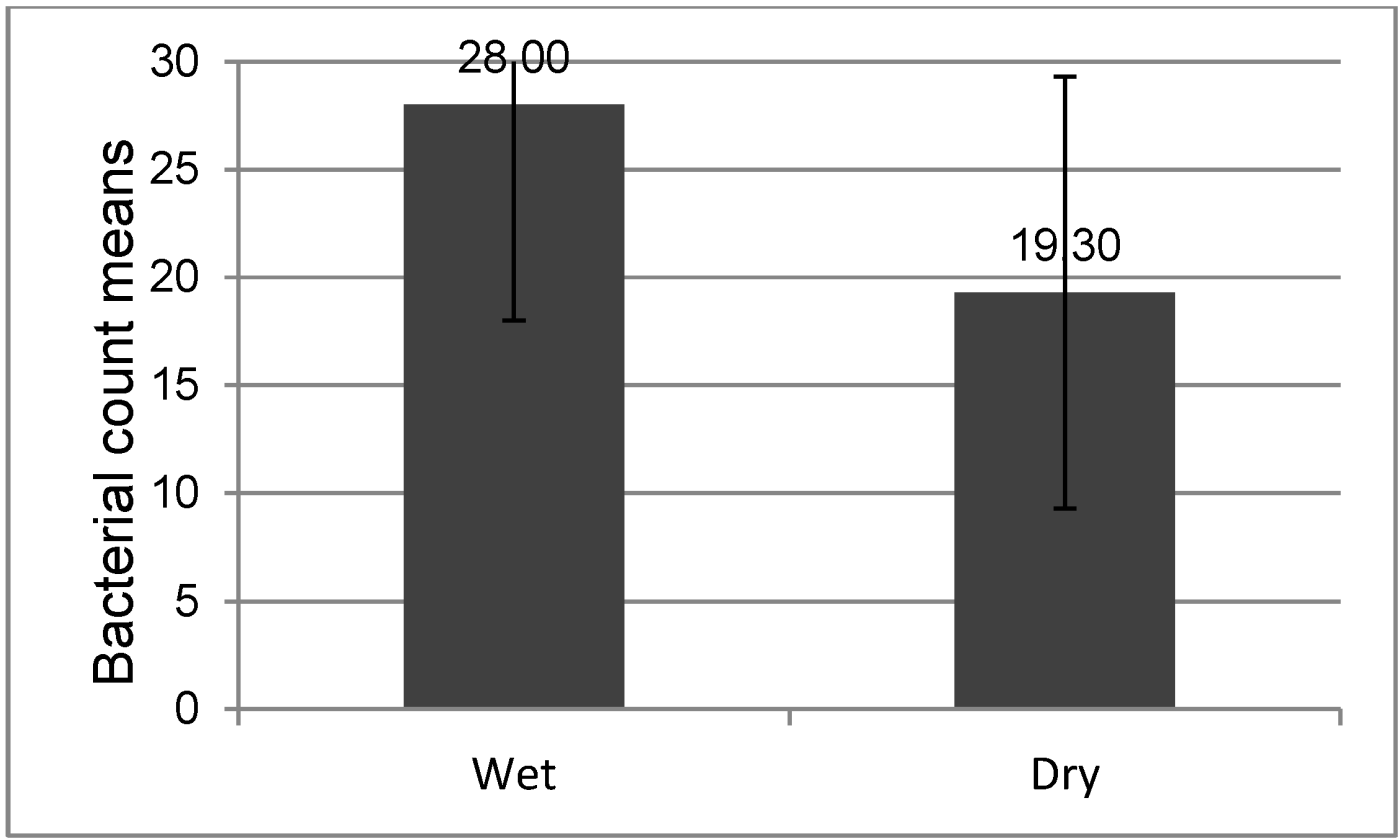
Comparison of *Bacillus subtilis* counts between the irradiated wet and dry cast groups

In the main experiment, microwave irradiation at a 900 W energy level was highly effective, as presented in Table 2 (Tab. 2). It shows a 6-log reduction in viability within 5 minutes, regardless of wet or dry conditions. The results of ANOVA and the t-test are depicted in Table 3 (Tab. 3) and Figure 5 (Fig. 5), and did not reveal significant differences in* B. subtilis* colony count between the dry and wet groups (P<0.05). Median CFU decreases of 6.31 and 6.67 log were observed in moist and dry conditions, respectively. 

In addition, the evaluation of gypsum specimens at 3x magnification did not reveal any holes, cracks, or color changes on the surface.

## Discussion

In spite of the high numbers of *B. subtilis* that were needed to show increased log reduction of microorganisms, the irradiation of contaminated dried and moistened stone casts resulted in a high level of disinfection. This striking 6-log decrease can be achieved in 5 minutes by a household microwave oven at 900 W, which microbicidally exceeds the requirements of EN 1040 standards [7].

In the pilot experiment, this level of disinfection was not achieved using 900 W in less than 15 minutes; however another study found that 850 W of microwave irradiation for 10 minutes resulted in sterilization with the stone cast immersed in water or a 40% concentration of NaCl solution [9]. This lethal effect on *B. subtilis* was previously achieved at a frequency of 2450 MHz with acrylic specimens, but using 650 W for 3 minutes under immersion in water [11]. Difference in effective energy levels and irradiation times in these experiments can be related to different strains of microorganisms and techniques used.

The current experiment showed no significant difference in the efficacy of microwave radiation exposure on moistened and dried stone casts. This was also observed in another study with different microorganisms at 600 W [10]. The moistened casts had been irradiated after removing from broth media as soon as the surface moisture had been absorbed in that and current studies. 

In addition to the non-thermal effects of microwaves that affect the biochemistry of cells and can inhibit or eliminate microorganisms [12], the thermal effect of microwaves is the other factor that results in killing the microorganisms. The thermal effect requires the presence of polar molecules such as water or ionic molecules [13], and makes bactericide possible at lower energy levels. Due to drying of the specimens during the first minutes of irradiation and the absence of water molecules (no thermal effect), the total elimination of microorganisms takes longer. As observed in the current pilot study and in a study by Abbas et al. [9], immersed specimens can be sterilized by 10 minutes of microwave irradiation, in comparison to the dry group that was only disinfected. 

The mechanical and physical properties of stone casts when irradiated without immersion in water or other liquids at 800–900 W for 5 minutes were not detrimentally affected [5], [6], [14], but immersion in water or 40% NaCl during the irradiation resulted in boiling the cast, negatively affecting the surface hardness and smoothness, as well as dimensional accuracy, as has been shown previously [9], [15].

The microorganism selected in the present study is one of the most resistant pathogens; it can convert into the spore form to tolerate harsh conditions. Among some typical indicator bacteria such as *Bacillus cereus*, *Staphylococcus aureus*, *Bacillus subtilis var. niger*, *Bacillus stearothermophilus*, *Bacillus pumilus E*, *B. subtilis* is considered to be an optimum indicator to evaluate the efficiency of microwave radiation [8].

It is recommended that further studies should be carried out with other strains of *B. subtilis* and other resistant species that can be used as indicator bacteria.

## Conclusions

Considering the conditions and limitations of the present study, it was shown that it is possible to disinfect casts contaminated with *B. subtilis* using microwave ovens. This technique can be useful in infection control, as it provides high-level disinfection of casts and dies. 

## Notes

### Competing interests

The authors declare that they have no competing interests.

### Acknowledgement

The authors are very grateful to the Research Vice Chancellor of Tabriz University of Medical Sciences for supporting this study. 

### Ethical considerations

All stages of this study were performed on a standard strain with in vitro conditions.

## Figures and Tables

**Table 1:**
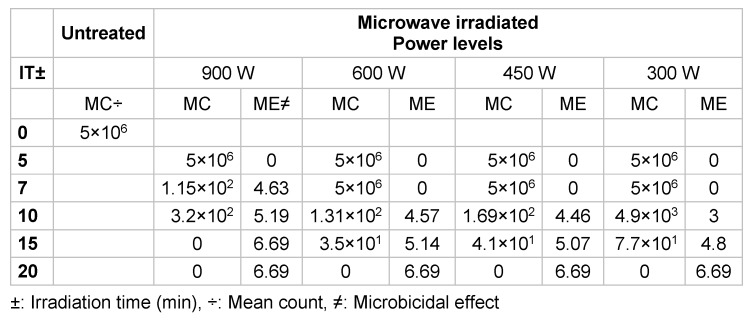
Mean counts (CFU/mL) and microbiocidal effect against *Bacillus subtilis* at different energy levels and irradiation duration

**Table 2:**
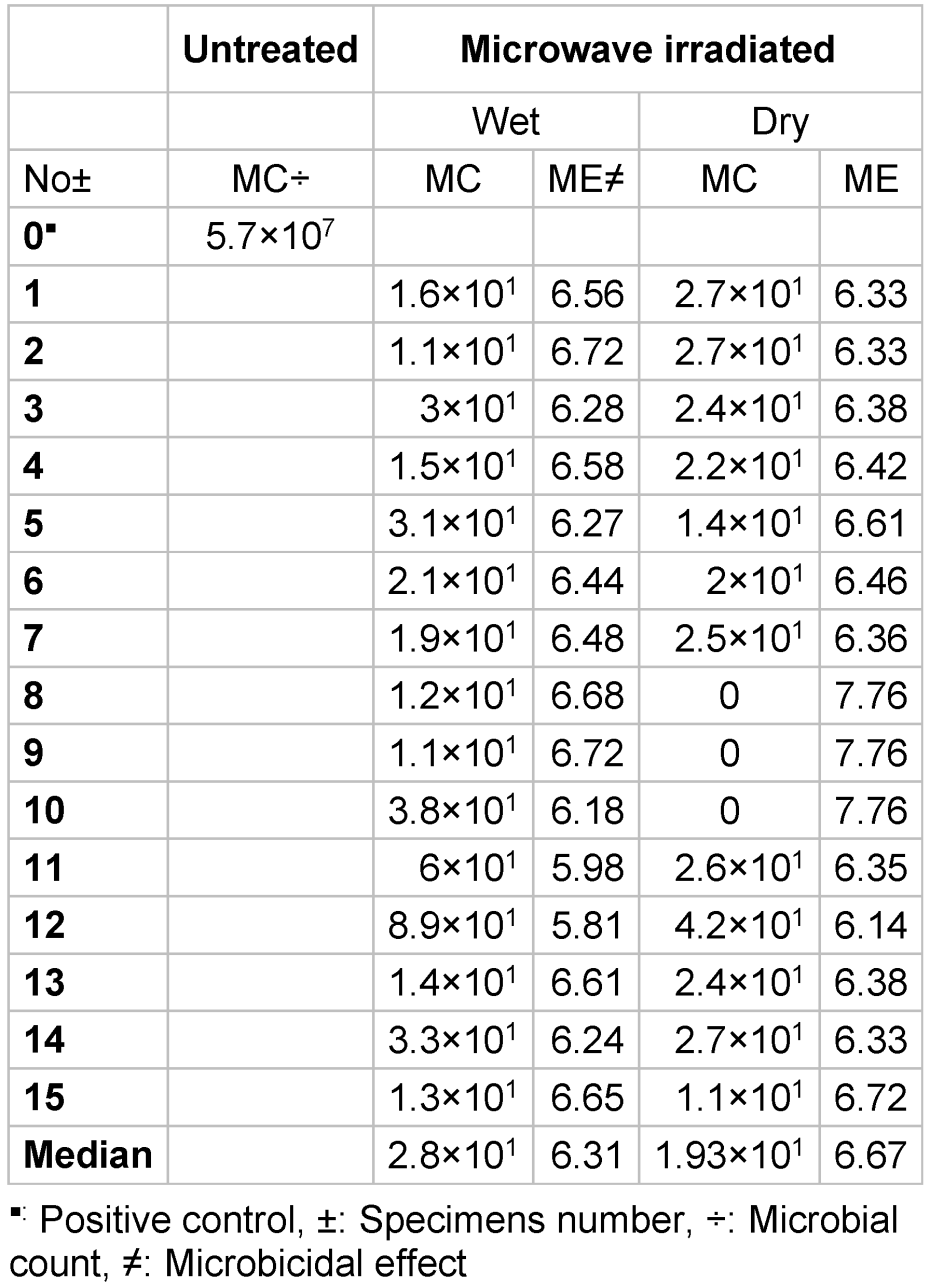
Colony count of *Bacillus subtilis* and microbicidal effect (ME) after irradiation of wet and dry casts at 900 W for 5 minutes

**Table 3:**
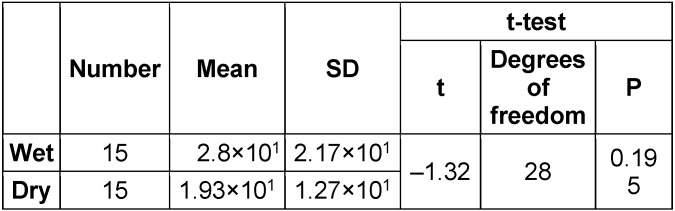
Results of ANOVA for *Bacillus subtilis* counts (CFU/mL) after irradiation in wet and dry cast conditions
